# A comparison of the effects of cinnamon, ginger, and metformin consumption on metabolic health, anthropometric indices, and sexual hormone levels in women with poly cystic ovary syndrome: A randomized double-blinded placebo-controlled clinical trial

**DOI:** 10.3389/fnut.2022.1071515

**Published:** 2022-11-29

**Authors:** Marsa Dastgheib, Reza Barati-Boldaji, Niki Bahrampour, Reihane Taheri, Mohammad Borghei, Sedigheh Amooee, Mohsen Mohammadi-Sartang, Alexei Wong, Siavash Babajafari, Seyed Mohammad Mazloomi

**Affiliations:** ^1^Nutrition Research Center, Shiraz University of Medical Sciences, Shiraz, Iran; ^2^Department of Nutrition, Science and Research Branch, Islamic Azad University (SRBIAU), Tehran, Iran; ^3^Metabolic and Cardiovascular Diseases Laboratory, Division of Human Nutrition, University of Alberta, Edmonton, AB, Canada; ^4^Department of Nutrition Sciences, School of Nutrition and Food Sciences, Tabriz University of Medical Sciences, Tabriz, Iran; ^5^Department of Gynecology and Obstetrics, School of Medicine, Shiraz University of Medical Sciences, Shiraz, Iran; ^6^Department of Health and Human Performance, Marymount University, Arlington, VA, United States; ^7^Department of Food Hygiene and Quality Control, School of Nutrition and Food Sciences, Shiraz University of Medical Sciences, Shiraz, Iran

**Keywords:** polycystic ovary syndrome, metformin, *Cinnamomum zeylanicum*, ginger, sexual hormones

## Abstract

**Background/objectives:**

Polycystic ovary syndrome (PCOS) is a prevalent endocrine disorder in women that can alter blood glucose, lipid profile and sexual hormonal level. Therefore, the evaluation of potential therapeutic agents in this population is important. The aim of the study was to determine the effects of cinnamon, ginger, and metformin intake on improvement of sex hormones level, metabolic health (lipid profiles, insulin level and fasting blood glucose) and anthropometric indices (weight, body mass index (BMI), etc.) in women with PCOS.

**Methods:**

A total of 100 women with PCOS were randomly assigned to one of the following four groups: cinnamon (500 mg of cinnamon, 3 × day), ginger (500 mg of ginger, 3 × day), metformin (500 mg of metformin 3 × day) or placebo. However, 17 participants were excluded for various reasons and consequently, 83 participants were considered for analysis. Sexual hormones, anthropometrics, glycemic and lipid markers were evaluated before and after the 8-week intervention.

**Results:**

Weight and BMI decreased significantly in all intervention groups. The consumption of metformin and cinnamon significantly decreased insulin resistance (HOMA-IR) in comparison to the placebo and ginger groups (*P* < 0.05). Moreover, a significant decrease in FSH (follicle-stimulating hormone) and LH (Luteinizing hormone) levels were observed in the ginger compared to the placebo group. While metformin and cinnamon significantly lowered testosterone levels (*P* < 0.05), none of the groups experienced a significant change in DHEA (dehydroepiandrosterone).

**Conclusion:**

Our findings suggest that in women with PCOS, cinnamon supplementation causes similar reductions in insulin resistance and testosterone level to that of metformin. Ginger supplementation decreased FSH and LH, hormonal effects not seen following metformin consumption. Overall, cinnamon and ginger supplementations may potentially be used as alternative treatment in women with PCOS.

**Clinical trial registration:**

[https://www.irct.ir/trial/28548], identifier [IRCT20171227038105N1].

## Introduction

Polycystic ovary syndrome (PCOS) is a hormonal imbalance affecting 4-8% of women in reproductive age that is characterized by hyperandrogenism, ovulatory dysfunction, and polycystic ovaries (1). Although the causes of PCOS are multifactorial and not fully understood, a combination of genetic and environmental factors have been shown to be associated to this condition (2, 3), including obesity, lack of physical exercise, family history and diabetes mellitus (4). Indeed, these factors are also associated to metabolic syndrome, which has a large prevalence in women with PCOS (∼30-40 %) (5). Metabolic abnormalities have a long term effects over the lifespan (6), and because the primary cause of PCOS is unknown, the treatment of this condition is directed at the improvement of both hormonal imbalance as well as metabolic parameters.

Pharmacological approaches for the treatment of PCOS include drugs for anovulation and androgenic symptoms as well as antidiabetic agents (especially metformin). Previous studies have demonstrated that metformin consumption causes beneficial metabolic and anthropometric effects in women with PCOS, including reductions in fasting blood glucose, triglyceride, total cholesterol, weight and body mass index (BMI) (7). However, conventional pharmacological management is limited by prevalence of contraindications, high costs and unwanted side-effects. Herbal medications represent an alternative therapy for improving and managing metabolic disorders in this population (8). While different herbal components have been proposed to aid in the treatment of PCOS, both ginger and cinnamon have recently emerged as plants of interest due to their anti-glycemic and anti-inflammatory properties (9).

Ginger (from the rizhome of Zingiber officinale Roscoe (family Zingiberaceae)) has been consumed worldwide as a flavoring agent and medicine for thousands of years. Previous studies indicate that ginger can improve dysmenorrhea and insulin resistance, reduce weight and inhibit ovarian cancer cells (10). Moreover, a prior investigation in rats with PCOS showed that 89 days of ginger extract consumption improved luteinizing hormone (LH), Follicle-stimulating hormone (FSH), estrogen and progesterone hormones (11). Additionally, Bonab et al. showed that 12 weeks of ginger supplementation decreased LH, testosterone and insulin levels in women with PCOS (12). Despite previous findings, evidence for the effect of ginger supplementation on hormonal balance, metabolic and anthropometric parameters in cohorts with PCOS is still inconsistent and unclear.

Cinnamon (*Cinnamomum zeylanicum*) is commonly used as a spice throughout the world. Prior research have revealed that cinnamon has a broad range of positive health effects, including hypoglycemic, anti-inflammatory, anti-lipidemic and antioxidant properties (13). Moreover, cinnamon is an herbal remedy used traditionally by patients with PCOS to improve their menstrual cycle (8). Although a small number of studies have investigated the effect of cinnamon supplementation on hormonal balance, metabolic and anthropometric parameters in cohorts with PCOS, the results are controversial (14).

There is little experimental evidence on the effects of ginger and cinnamon supplementation on sex hormones level, metabolic health and anthropometric indices in women with PCOS. On the other hand, there is no study in cohorts with PCOS comparing the effect of metformin to herbal supplements (such as ginger and cinnamon) on the aforementioned parameters. Hence, we compared the effect of ginger, cinnamon and metformin on the improvement in biochemical biomarkers including sex hormones level, glycemic control, lipid profile, anthropometric indices in women with PCOS. We hypothesized that ginger, cinnamon and metformin intake would improve our primary outcomes of biochemical biomarkers (including sex hormones levels), glycemic control and lipid profile, as well as the secondary outcomes measures of anthropometric indices.

## Materials and methods

### Participants

A total of 100 women with PCOS (age: 29 ± 6 years) were recruited from the Shahid Motahhari endocrinology clinic of Shiraz, Iran. The inclusion criteria were as follows: diagnosis of PCOS by a co-advisor endocrinologist based on ESHRE-ASRM criteria (15), age 20 to 40 years old, no use of hormonal drugs for the treatment of menstrual disorders in the past three months, no history of allergies to cinnamon and ginger, and consent for participation. Exclusion criteria were as follows: menopause, hyperlipidemia [total cholesterol (TC) > 240 mg/dL (6.21 mmol/L), low-density lipoprotein (LDL) > 100 mg/dl, high-density lipoprotein (HDL) < 50 mg/dL (1.3 mmol/L) and triglycerides (TG) > 150 mg/dl], diabetes, hyperprolactinemia, thyroid disorders, changes in physical activity levels in the past 6 months, taking any medicines including antihyperglycemic agents, lipid lowering medications, contraceptives, corticosteroids, and use of any supplements other than those provided for in the study. Other than their assigned intervention, the participants were asked not to change their lifestyle (including the maintenance of their current physical activity level) throughout the length of the investigation. The present research was performed based on the guidelines of declaration of Helsinki. The study protocol was approved by the ethics committee of Shiraz University of Medical Sciences, Shiraz, Iran, and was registered in the Iranian Registry of Clinical Trials (IRCT20171227038105N1). The study protocol was explained to all participants, which subsequently read and signed an informed consent form before study enrollment. The study sample size was calculated by accounting for a type I error of 5, 95% confidence interval, ∂ = 0.2, ε = 0.1, and α = 0.05 and statistical power of 80% in detecting 3–5% variability between the intervention groups vs. control on our primary study outcome of total testosterone. The final sample was estimated including a 20% probable drop-out for each group. A minimum detectable effect size (i.e., Δ of lab endpoints) of 0.3 was considered clinically plausible using data from previous investigations utilizing ginger and cinnamon supplementations (9, 11, 16–18). Our calculated sample size was 80 participants (20 participants per group).

### Study design

In this four−arm, double-blinded, randomized placebo-controlled trial, 100 eligible participants were recruited into a 2-week run-in period to obtain detailed information about their dietary intake and physical activities. The participants were then randomly assigned into one of the following four groups: Placebo Group, Ginger Group, Cinnamon Group and a Metformin Group for a period of 8 weeks. A computer-generated allocation list of random permuted blocks was used to ensure concealment of allocation to subjects and investigators. Emergency code-break envelopes were retained with first author, but no code breaking was required during the study. A block randomization method (Excel software) with block size of 4 was used for group assignment. The random function was be used to generate random sequence within each block. After determining the allocated intervention, a non-repetitive four-digit random code was assigned to each participant. Assigned codes were delivered to the eligible participants via text messages. Before the beginning of the investigation, participants became familiar with all testing protocols and procedures. Hormone level, glycemic control, lipid profile, anthropometric indices, physical activity and dietary intake were assessed before and after the 8-week study. Moreover, measurements were performed in follicular phase of the menstrual cycle, and random days in women with oligo/amenorrhea (19). The participants and researchers were blinded to the dietary assignment. Compliance to study protocol and potential adverse effects were assessed by biweekly via phone call. The participants were instructed not to change their lifestyle or habitual dietary intake patterns throughout the length of the investigation.

### Supplementations

Participants in Placebo Group ingested a rice flour capsule as a placebo which were prepared by the Faculty of Pharmacy, Shiraz University of Medical Sciences. Participants in the Ginger Group ingested capsules containing 500 mg of Zingiber officinale Roscoe powder, while those in Cinnamon Group receiving capsules containing 500 mg of *Cinnamomum zeylanicum* extract as the active ingredient. Cinnamon and ginger powder were provided from the Iranian Institute of Medicinal Plants, Tehran, Iran. The Metformin Group consumed 500 mg of metformin hydrochloride enteric-coated capsules manufactured by Aria Pharmaceutical Company (approval number: 9609356). All participants consumed their assigned capsules 3 times per day, before breakfast, lunch and dinner. All capsules were of the same size, shape and color. The selected dose and time of ingestion were based on prior investigations (20). Supplementation compliance was estimated by dividing the consumed capsules by the expected number of capsules.

### Measurements

All data were collected by trained researchers. Information about age, medical history, current use of medications, and cigarette smoking were obtained by face-to-face interview. Participant’s height was measured via a wall-mounted stadiometer, to the nearest 0.5 cm. Weight was established with participants dressed in light clothes and no shoes, using a digital scale to the nearest 0.1 kg. BMI was calculated as weight (kg) divided by the square of height (m^2^). Waist circumference (WC) was measured at the level of the iliac crest at the end of normal expiration to the nearest 0.5 cm (21). Physical activity was determined using the international physical activity questionnaire (IPAQ) (22), while dietary intake was assessed by a 24-h food recall (23). The content of energy and nutrients were estimated using NUTRITIONIST IV software (24).

5-cc venous blood samples were taken from each participant in the morning, after 10-12 h of fasting. Blood samples were distributed among tubes containing K2EDTA then centrifuged to separate serums. The serums were stored at −72°C for further biochemical analysis. Fasting blood sugar (FBS) was quantified via the glucose oxidase/peroxidase method using standard kits (Pars Azmoon Inc., Tehran, Iran). Glycated hemoglobin (HbA1c) percentage was quantitatively estimated in vitro using a Dimension^®^ system (Dade Behring Inc., Milton Keynes, UK). TC, LDL, HDL, and TG concentrations were assayed using standard kits (Pars Azmoon Inc., Tehran, Iran). Serum insulin level, LH, FSH, testosterone, DHEA and sex hormone binding globulin (SHBG), was measured by enzyme-linked immunosorbent assay (ELISA) using standards kit (MONOBIND INC., US). In all the biochemical analyses, the intra-assay and inter-assay coefficients of variation (CVs) were less than 2.0% and < 15%, respectively.

Free androgen index (FAI) was calculated as the ratio of total testosterone level to SHBG values (25). Insulin resistance was determined by HOMA-IR index that was calculated using the following formula (26):


HOMA-IR=Fasting⁢blood⁢glucose⁢mg/dl×Serum⁢insulin⁢level⁢μ⁢U/ml/405

All the laboratory processes were performed in the laboratory of Nutrition and Food Science School, Shiraz University of Medical Sciences, under the supervision of qualified experts and carried out under standard laboratory conditions.

### Statistical analysis

Data analyses were conducted using the Statistical Package for Social Sciences (version 16.0; SPSS Inc.). Shapiro Wilk test was used to check data normality. Accordingly, baseline characteristics of participants were compared through one-way analysis of variances (ANOVA). We compared the variables before and after 8 weeks of intervention between groups through generalized linear model. Significant interactions or main effects were followed up using Bonferroni post hoc analyses. Significant interactions or main effects were followed up using Bonferroni post hoc analyses. The results with *P* value lower than 0.05 was considered as statistically significant.

## Results

### Participant flow

**FIGURE 1:**
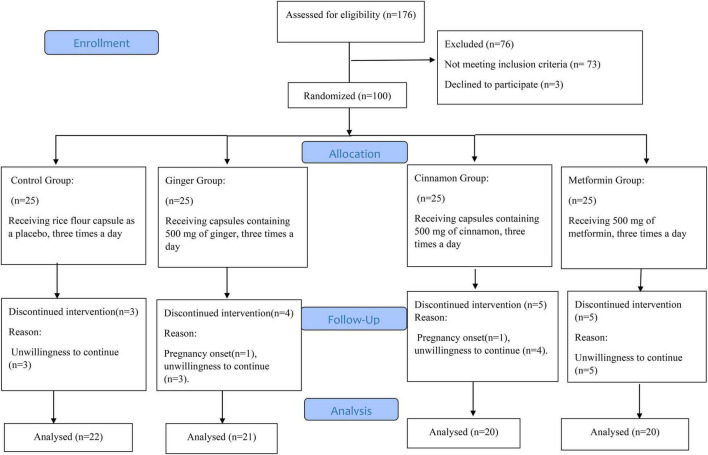
The CONSORT flow diagram.

The participant flow diagram is displayed in Figure 1. The study was performed between August and December of 2017. We screened 176 women with PCOS. 100 of these qualified for baseline evaluation. After randomization, two participants were excluded for becoming pregnant (Ginger Group = 1, Cinnamon Group = 1) and fifteen for unwillingness to continue in the study (Placebo Group = 3, Ginger Group = 3, Cinnamon Group = 4, and Metformin Group = 5). Consequently, 83 of the 100 women with PCOS included completed the trial and were analyzed as per protocol. Data are presented for the 83 participants that successfully completed our 8-week intervention. Supplementation compliance was >90% in all groups. Moreover, no adverse events were reported throughout the study.

### Baseline data

The baseline characteristics of participants are shown in Table 1. Baseline values were similar in all groups, as no significant differences were established (all *P*-values > 0.05). Table 2 shows the macronutrient, energy intake and physical activity levels before and after 8 weeks. No significant difference was seen in the dietary intake and physical activity levels of the patients before and after intervention (*p*-value > 0.05).

**TABLE 1:** Baseline characteristics of the participants.

Variable	Group A: Flour capsule -placebo (*n* = 22)	Group B: Ginger (*n* = 21)	Group C: Cinnamon (*n* = 20)	Group D: Metformin (*n* = 20)	*P*-value*
Age (year)	30.00 ± 6.37	28.45 ± 4.96	30.20 ± 5.72	29.95 ± 7.07	0.77
Weight (kg)	71.80 ± 13.46	65.10 ± 12.48	72.70 ± 12.31	71.80 ± 12.14	0.20
BMI (kg/m2)	27.52 ± 5.09	26.14 ± 4.99	28.61 ± 4.65	27.76 ± 4.63	0.31
FBS (mg/dl)	102.70 ± 17.09	100.55 ± 22.36	100.85 ± 18.54	94.00 ± 14.40	0.35
Insulin (μU/ml)	7.51 ± 3.93	7.10 ± 4.10	8.12 ± 5.94	8.38 ± 3.61	0.69
HOMA-IR	1.89 ± 0.97	1.80 ± 1.16	2.15 ± 1.91	1.98 ± 0.92	0.66
TG (mg/dl)	159.20 ± 58.27	163.10 ± 95.01	150.15 ± 39.82	161.65 ± 63.27	0.77
TC (mg/dl)	174.50 ± 36.85	171.10 ± 37.40	190.70 ± 33.44	175.45 ± 27.94	0.16
LDL-C (mg/dl)	97.70 ± 25.18	90.40 ± 28.50	106.80 ± 21.56	89.30 ± 23.31	0.06
HDL-C (mg/dl)	32.50 ± 7.76	33.70 ± 4.51	36.65 ± 4.74	33.30 ± 4.09	0.09

Values for continuous variables, as mean ± standard deviation with normal distribution.

*Baseline parameters are compared using one-way ANOVA. *P* < 0.05 is considered significant.

**TABLE 2:** Macronutrients, energy, cholesterol and fiber intake and physical activity level before and after intervention.

Variable		Group A: Flour capsule -placebo (*n* = 22)	Group B: Ginger (*n* = 21)	Group C: Cinnamon (*n* = 20)	Group D: Metformin (*n* = 20)	*P*-value**
Energy (kcal/d)	Before	1673.73 ± 583.45	1592.41 ± 427.29	1567.43 ± 532.16	1646.82 ± 803.32	0.47
	After	1548.33 ± 458.32	1652.79 ± 468.31	1576.5 ± 413.37	1584.68 ± 416.41	
	*P*-value*	0.13	0.41	0.58	0.24	
Carbohydrate (g/d)	Before	253.68 ± 100.13	251.1 ± 101.33	239.53 ± 95.83	249.11 ± 102.33	0.13
	After	232.51 ± 88.96	247.68 ± 101.13	264.36 ± 84.89	234.73 ± 96.66	
	*P*-value*	0.18	0.37	0.49	0.63	
Protein (g/d)	Before	56.43 ± 37.42	53.04 ± 31.14	52.31 ± 19.33	53.00 ± 35.19	0.15
	After	48.68 ± 32.31	54.48 ± 33.62	53.89 ± 16.13	50.49 ± 23.09	
	*P*-value*	0.09	0.65	0.89	0.45	
Fat (g/d)	Before	52.34 ± 30.48	48.71 ± 29.11	48.19 ± 21.96	47.66 ± 28.1	0.69
	After	43.78 ± 27.81	51.33 ± 30.63	39.87 ± 24.55	52.29 ± 30.68	
	*P*-value*	0.08	0.23	0.10	0.34	
Cholesterol (mg/d)	Before	168.34 ± 153.21	201.83 ± 183.68	192.76 ± 133.13	207.80 ± 183.69	0.35
	After	153.76 ± 85.93	169.28 ± 151.15	160.81 ± 79.96	181.73 ± 49.40	
	*P*-value*	0.24	0.21	0.18	0.20	
Fiber (g/d)	Before	12.16 ± 5.13	11.38 ± 4.49	14.51 ± 7.11	13.17 ± 4.51	0.34
	After	13.19 ± 5.13	11.13 ± 4.07	14.33 ± 5.13	11.15 ± 5.33	
	*P*-value*	0.74	0.81	0.84	0.11	
Physical activity (MET. min/week)	Before	932.5 ± 402.6	438.4 ± 234.5	912.8 ± 343.9	898.8 ± 345.6	0.41
	After	968.86 ± 512.7	1012.7 ± 340.1	1102.53 ± 449.5	1076 ± 225.3	
	*P*-value*	0.68	0.11	0.14	0.22	

Values for continuous variables, as mean ± standard deviation.

*Within group comparison.

**Before-Baseline parameters are compared using one-way ANOVA. *P* < 0.05 is considered significant.

MET, Metabolic Equivalent.

### Hormonal levels

Table 3 describes the effect of ginger, cinnamon and metformin on sexual hormones before and after the 8-week intervention. Post hoc analysis indicated that consumption of ginger decreased the FSH in comparison to the placebo group. No other significant changes were observed in FSH levels. Consumption of ginger significantly decreased LH compared to placebo [6.6 vs. 9.1 mIu/ml (95% CI, 5.49 to 7.83 vs. 6.12 to 12.08; *P* = 0.02]. FSH level was also decreases in mentioned groups. Significant reductions in testosterone levels were observed after metformin [49.5 vs. 61.05 ng/dl (95% CI, 36.61 to 62.39 vs. 49.65 to 72.45; *P* = 0.01] and cinnamon consumption compared to placebo [50.05 vs. 61.05 nmol/l (95% CI, 38.41 to 61.68 vs. 49.65 to 72.45; *P* = 0.01]. None of the groups showed a significant change in DHEA.

**TABLE 3:** Sexual hormones level of the patients before and after 8 weeks intervention.

Variable		Group A: Flour capsule -placebo (*n* = 22)	Group B: Ginger (*n* = 21)	Group C: Cinnamon (*n* = 20)	Group D: Metformin (*n* = 20)	*P*-value**
FSH (mIu/ml)	Before	8.15 ± 8.57	5.53 ± 1.56	8.3 ± 3.73	8.61 ± 8.54	**0.02**
	After	10.02 ± 13.09	4.29 ± 1.56 **^a^**	8.09 ± 6.93	12.46 ± 21.03 **^c^**	
	*p*-value*	0.19	**0.006**	0.37	0.10	
LH (mIu/ml)	Before	13.91 ± 15.5	19.47 ± 20.44	12.72 ± 6.56	11.02 ± 6.03	**0.02**
	After	9.1 ± 9.36	6.66 ± 2.5	10.88 ± 4.87 **^b^**	14.67 ± 16.12**^b,c^**	
	*p*-value*	0.17	**0.001**	0.26	0.82	
Testosterone (ng/dl)	Before	65.07 ± 26.61	61.15 ± 20.46	65.8 ± 29.63	62.6 ± 32	**0.01**
	After	61.05 ± 24.36	62.1 ± 26.03**^c,d^**	50.05 ± 24.86 **^a^**	49.5 ± 27.54	
	*p*-value*	0.39	0.82	** < 0.001**	**0.001**	
DHEA (ng/ml)	Before	1845.6 ± 1197.92	1682.6 ± 897.11	2079 ± 1209.21	2842.8 ± 2846.67	0.37
	After	1890.6 ± 1332.34	1719.7 ± 814.62	1862.5 ± 1020.94	2491.4 ± 1922.58	
	*p*-value*	0.85	0.65	0.11	0.20	
SHBG (nmol/L)	Before	51.9 ± 26.02	63.27 ± 29.85	45.85 ± 27.47	41 ± 15.54	0.16
	After	47.9 ± 22.49	55.55 ± 21.01	52.22 ± 33.69	46.36 ± 28.41	
	*p*-value*	0.55	**0.03**	0.16	0.97	
FAI	Before	1.68 ± 1.41	1.24 ± 0.87	2.28 ± 2.15	1.92 ± 1.42	0.22
	After	1.71 ± 1.45	1.26 ± 0.96	1.88 ± 2.3	1.42 ± 1.14	
	*p*-value*	0.79	0.47	**0.03**	0.31	

Values for continuous variables, as mean ± standard deviation.

*Within group comparison.

**Before-Baseline parameters are compared using one-way ANOVA. *P* < 0.05 is considered significant (*P*-value numbers marked in bold indicate numbers that are significant).

^a^Significant difference compare to group A (*p* < 0.05).

^b^Significant difference compare to group B (*p* < 0.05).

^c^Significant difference compare to group C (*p* < 0.05).

^d^Significant difference compare to group D (*p* < 0.05).

FSH, Follicle-Stimulating Hormone; LH, Luteinizing Hormone; DHEA, Dehydroepiandrosterone; SHBG, Sex Hormone Binding Globulin; FAI, Free Androgen Index.

### Anthropometric and metabolic parameters

After 8 weeks, body weight and BMI decreased significantly in all intervention groups (*p*-values <0.05) while no significant change was seen in placebo group (*p*-value > 0.05). No significant differences (*P*-values > 0.05) in weight and BMI decrements were observed between the intervention groups (ginger, cinnamon and metformin). Consumption of metformin significantly decreased insulin levels when compared to placebo [6.84 vs. 8.31 μU/ml (95% CI, 5.83 to 7.85 vs. 4.89 to 11.73; *P* = 0.01]. Both ginger and cinnamon groups had no significant effects on insulin level (Table 4). Lipid profiles indices (TG, TC, LDL, and HDL) showed no significant changes in all groups except for TC, which decreased in the metformin group from 175.45 ± 27.94 to 160.20 ± 22.92 (*P*-value = 0.008). Furthermore, cinnamon and metformin significantly decreased insulin resistance (HOMA-IR) in comparison to the placebo group [1.25 vs. 1.93 (95% CI, 1.10 to 1.96 vs. 1.56 to 2.29; *P* = 0.04], [1.58 vs. 1.93 (95% CI, 1.34 to 1.82 vs. 1.56 to 2.29; *P* = 0.04] respectively.

**TABLE 4:** Lipid profile, anthropometric and glycemic indices of the patients before and after intervention.

Variable		Group A: Flour capsule -placebo (*n* = 22)	Group B: Ginger (*n* = 21)	Group C: Cinnamon (*n* = 20)	Group D: Metformin (*n* = 20)	*P*-value**
Weight (kg)	Before	71.8 ± 13.4	65.1 ± 12.48	72.7 ± 12.31	71.8 ± 12.14	0.132
	After	71.05 ± 12.16	63.77 ± 10.82	70.65 ± 10.79	69.15 ± 11.40	
	*p*-value *	0.167	**0.021**	**0.02**	**0.02**	
BMI (kg/m^2^)	Before	27.52 ± 5.09	26.14 ± 4.99	28.61 ± 4.6	27.76 ± 4.63	0.265
	After	27.23 ± 4.54	25.61 ± 4.24	27.82 ± 4.21	26.75 ± 4.27	
	*p*-value*	0.136	**0.044**	**0.04**	**0.04**	
WC (cm)	Before	82.4 ± 14.44	81.5 ± 13.59	86.35 ± 15.67	84.25 ± 15.98	0.898
	After	80.7 ± 12.92	80.45 ± 12.68	84.5 ± 14.26	82.80 ± 16.01	
	*p*-value*	**0.026**	**0.024**	**0.09**	**0.04**	
WHR	Before	0.77 ± 0.08	0.76 ± 0.06	0.79 ± 0.09	0.78 ± 0.08	0.951
	After	0.76 ± 0.08	0.75 ± 0.05	0.77 ± 0.07	0.77 ± 0.09	
	*p*-value*	**0.037**	0.121	**0.011**	**0.024**	
FBS (mg/dl)	Before	102.70 ± 17.09	100.55 ± 22.36	100.85 ± 18.54	94.00 ± 14.40	0.6
	After	94.15 ± 7.42	98.40 ± 15.73	93.45 ± 17.09	93.90 ± 9.13	
	*P* value*	**0.04**	0.35	0.10	0.76	
Insulin (μU/ml)	Before	7.51 ± 3.93	7.10 ± 4.10	8.12 ± 5.94	8.38 ± 3.61	**0.01**
	After	8.31 ± 3.42	6.33 ± 3.75	6.23 ± 2.97	6.84 ± 2.15 **^a^**	
	*P* value*	0.24	0.31	0.05	**0.006**	
HOMA-IR	Before	1.89 ± 0.97	1.80 ± 1.16	2.15 ± 1.91	1.98 ± 0.92	**0.04**
	After	1.93 ± 0.77	1.58 ± 1.07	1.53 ± 0.92 **^a,b^**	1.58 ± 0.51 **^a,b^**	
	*P* value*	0.40	0.22	**0.01**	**0.01**	
TG (mg/dl)	Before	159.20 ± 58.27	163.10 ± 95.01	150.15 ± 39.82	161.65 ± 63.27	0.56
	After	133.90 ± 36.79	128.70 ± 38.82	155.50 ± 65.73	154.50 ± 50.95	
	*P* value*	0.07	0.24	0.88	0.55	
TC (mg/dl)	Before	174.50 ± 36.85	171.10 ± 37.40	190.70 ± 33.44	175.45 ± 27.94	0.41
	After	167.40 ± 35.94	171.20 ± 42.94	180.00 ± 27.46	160.20 ± 22.92	
	*P* value*	0.08	0.95	0.17	**0.008**	
LDL (mg/dl)	Before	97.70 ± 25.18	90.40 ± 28.50	106.80 ± 21.56	89.30 ± 23.31	0.32
	After	95.15 ± 20.34	95.80 ± 23.10	101.25 ± 31.81	85.45 ± 18.35	
	*P* value*	0.43	0.38	0.09	0.525	
HDL (mg/dl)	Before	32.50 ± 7.76	33.70 ± 4.51	36.65 ± 4.74	33.30 ± 4.09	0.78
	After	35.20 ± 4.40	34.35 ± 5.20	38.85 ± 8.56	35.95 ± 5.33	
	*P* value*	0.13	0.64	0.29	0.07	

Values for continuous variables, as mean ± standard deviation.

*Within group comparison.

**Before-Baseline parameters are compared using one-way ANOVA. *P* < 0.05 is considered significant (*P*-value numbers marked in bold indicate numbers that are significant).

^a^Significant difference compare to group A (*p* < 0.05).

^b^Significant difference compare to group B (*p* < 0.05).

BMI, Body Mass Index; WC, Waist Circumference; WHR, Waist to Hip Ratio; FBS, Fasting Blood Sugar; HOMA-IR, Homeostatic Model Assessment of Insulin Resistance; TG, Triglyceride; TC, Total cholesterol; LDL-C, low-density lipoproteins; HDL-C, high-density lipoproteins.

## Discussion

Our randomized double-blinded placebo-controlled clinical trial aimed to compare the effects of ginger, cinnamon and metformin on insulin resistance, lipid profile, anthropometric markers, and sexual hormones in women with PCOS. It is important to find alternative strategies for the treatment of PCOS as consumption of metformin may cause discomfort and side effects (27). Cinnamon supplementation causes similar reductions in body weight, insulin resistance testosterone and FAI to that of metformin. Ginger supplementation decreased FSH, LH and SHBG, hormonal effects not seen following metformin consumption. Following 8 weeks of intervention, body weight and BMI decreased significantly in all intervention groups. Additionally, cinnamon supplementation led to similar declines in body weight, insulin resistance (HOMA-IR), testosterone and FAI to that of metformin. Furthermore, ginger supplementation decreased the FSH, LH, and SHBG concentrations in women with PCOS.

### Sexual hormones

Hyperandrogenemia has a key role in pathogenesis of PCOS. There is an association between insulin resistance and hyperinsulinemia with androgen excess. Hyperinsulinemia increases androgen production (by stimulating ovarian steroidogenesis) and inhibits sex hormone-binding globulin (SHBG) production. At higher concentrations, insulin binds to the insulin-like growth factor (IGF) receptors, transmits its signals to ovarian hyperandrogenic state as a consequence of an enzymatic dysregulation of P450c17α, and inhibits hepatic SHBG synthesis. This reduction leads to greater levels of free androgens in blood (28).

In this study, consumption of Ginger decreased FSH, LH and SHBG level in women with PCOS. In line with present study, Atashpour et al. showed that high dose of ginger extract, has improving effects in balancing LH, FSH, estrogen and progesterone hormones in rats with PCOS (11). Hormonal changes including elevated levels of androgens (testosterone, DHEA and androstenedione), estrogens, and LH and reduced FSH levels are seen in women with PCOS. The decreasing level of SHGB in this study may be caused by insignificance difference in insulin level in ginger group (29). Perhaps, lowering these hormones level reduce follicular arrest accumulation of small follicles (29). Consumption of Metformin and Cinnamon in patients with PCOS caused a reduction in testosterone level. Additionally, FAI was reduced significantly in cinnamon group while other groups showed no difference. It appears that cinnamon can mimic the metformin effect on testosterone level. In agreement with our findings, Hajimonfarednejad et al. showed a reduction in testosterone levels after cinnamon supplementation (1.5 g/d for 12 weeks) in women with PCOS (8). On the contrary, Kort D et al. demonstrated that consumption of 1.5 g/d cinnamon for 6 months in 45 women with PCOS did not change total testosterone, DHEA, SHBG (30). As there is a clear relationship between insulin resistance and LH and testosterone as an androgen (31), it seems reduction of insulin resistance by cinnamon and metformin in the current study, caused a further reduction of testosterone an FAI level.

### Glycemic indices and anthropometrics

Insulin resistance is suggested as a key factor in etiology of PCOS. A two-way relationship exists between metabolic syndrome and PCOS, with a large proportion of women with PCOS suffering from metabolic syndrome (32). Furthermore, there is also correlation between PCOS and excess body weight, as women with PCOS also have an increased prevalence of weight gain and overweight and obesity. Guidelines stated that the first line treatment of PCOS should include weight management via lifestyle changes, such as the use of dietary interventions (33).

A significant reduction in weight and BMI was seen after 8 weeks in all intervention groups of intervention. However, there were no significant differences between the intervention groups in these markers. Since ginger and cinnamon can cause a weight reduction in the equal dimension to metformin, it seems they can play the alternative role of Metformin in weight reduction. In line with this study, a previous systematic review and meta-analysis of controlled clinical trials revealed that ginger intake significantly reduces effect on weight and waist to hip ratio (WHR) in overweight and obese individuals (34). Interestingly, another systematic review and meta-analysis of controlled clinical trials showed that cinnamon supplementation significantly reduces body weight (mean difference of −0.92 kg), BMI (mean difference of −0.40 kg/m^2^), and waist to hip ratio (WHR) in adults (35). Moreover, Borzoei et al. found a significant reduction in BMI and weight in women with PCOS after 8 weeks of cinnamon supplementation with 1500 mg/day (similar dose to the current study) (36). Importantly, our findings indicate that cinnamon and ginger supplementation have similar weight-loss effects to that of metformin in women with PCOS, a medication used for insulin resistance and weight reduction (31).

Insulin resistance is highly prevalence in patients with PCOS (1). HOMA-IR, an indicator of insulin resistance, was also significantly decreased by metformin and cinnamon intake. Moreover, no difference was seen in the ability of cinnamon and metformin in reducing HOMA-IR. As a result, cinnamon seems to be as effective as metformin in increasing the Insulin sensitivity. In line with this study, Maleki et al. showed that HDL and insulin sensitivity were increased by the cinnamon supplementation while LDL, TG, and blood glucose were decreased in patients with PCOS (17). Supplementation with cinnamon has direct connection with lowering oxidative stress, leptin, total energy expenditure and liver enzymes, factors that may lead to an improvement in HOMA-IR (37).

### Lipid profile

In relation to lipid profile, TG, HDL, and LDL did not change in intervention groups while TC was decreased only in the metformin group. Khan et al. showed that consumption of either 1, 3 or 6 grams of cinnamon in patients with type 2 diabetes for 40 days can decrease LDL and TC levels significantly (38), while in the study conducted by Hajimonfarednejad et al. after 12 weeks of supplementation with 1,500 mg cinnamon in women with PCOS, HOMA-IR level decreased but no significant change in TC level was seen (8). In line with this investigation, there were no significant changes in mean of serum TC, LDL-C, HDL-C levels following 10 weeks of ginger supplementation in peritoneal dialysis patients (39).

### Potential mechanisms

Cinnamon and its active ingredients such as cinnamaldehyde, cinnamate, cinnamic acid, and eugenol in the forms of aqueous and alcoholic extracts have anti-obesity effect through reduction of Ghrelin, insulin resistance, lipolysis, lipogenesis, lipid absorption in the small intestine (40). Cinnamon polyphenols (CP) may have an insulin-like activity in cells (41) and activates insulin receptor by increasing their tyrosine phosphorylation activity and by decreasing phosphatase activity that inactivates the insulin receptor (41).

The insulin-like biological and Kinase inhibition activity is likely related to polyphenol type A polymers present in Cinnamon. Purified components of Cinnamon, procyanidin type A trimers and a tetramer, were found to enhance insulin activity, glucose uptake and glycogen synthesis and mRNAs for the insulin receptor and glucose transporter 4 (GLUT4). Cinnamaldehyde, the major component of Cinnamon, up regulates the expression of GLUT4 and act as an insulin mimetic by stimulating the translocation of GLUT4 in a Phosphoinositide 3-kinases (PI3K) dependent manner (42).

Although some studies reported anti-diabetic, anti-hyperlipidemia and anti-obesity effect of ginger (43), in this study ginger only could decrease weight and BMI with no significant effect on FBS, insulin resistance and lipid profile. Ginger could (1) increasing thermogenesis and energy expenditure through catecholamine-releasing action, (2) increasing the lipolysis of white adipose tissue and (3) inhibition of the lipase enzyme and the intestinal absorption of dietary fat (10). The result of these mechanisms is decreasing weight and body fat.

### Study strengths and limitations

The strengths of the present study include its double-blind placebo-controlled design. Moreover, this is the first study comparing effect of ginger, cinnamon and metformin on metabolic health, anthropometrics and sexual hormones in women with PCOS. Pharmacological therapy is the treatment of choice in women with PCOS, which includes metformin and oral contraceptives. However, this line of treatment is limited by discomfort and side effects in this population (27). Therefore, alternative non-pharmacological strategies add more options for patients. Limitations of the current study include the lack of assessment of free testosterone, DHEAS, clinical complications like alopecia, hirsutism, irregular cycles as well as the long-term effects of ginger and cinnamon intake like infertility. Moreover, not all our participants had IR and elevated testosterone; including only participants with these conditions would have probably rendered a stronger interventional response. Our investigation is also limited by its short length and fixed supplementation dose. Consequently, we cannot generalize our result to other cohorts and we suggest the evaluation of longer treatment periods and different dosages in future studies. Furthermore, investigations focusing on bleeding patterns, hirsutism and fertility would be more practical for the clinician.

## Conclusion

Our findings suggest that in women with PCOS, cinnamon supplementation causes similar reductions in body weight, insulin resistance, testosterone level to that of metformin. Ginger supplementation decreased FSH, LH and SHBG, hormonal effects not seen following metformin consumption. Overall, cinnamon and ginger supplementations might be used as alternative treatment in women with PCOS. Future investigations should evaluate the combined consumption of metformin with these herbal supplements to establish if our observed improvements can be amplified. It is important to find alternative strategies for the treatment of PCOS as consumption of metformin may cause discomfort and side effects (27).

## Data availability statement

The raw data supporting the conclusions of this article will be made available by the authors, without undue reservation.

## Ethics statement

The study protocol was approved by the Ethics Committee of Shiraz University of Medical Sciences (IR.SUMS.REC.1396.41, dated 2017-06-11). The clinical trial is registered with the Iranian Registry of Clinical Trials (IRCT20171227038105N1, dated 2018-01-30) Shiraz, Iran. The patients/participants provided their written informed consent to participate in this study.

## Author contributions

MD and SB: contribution to the conception and design of the work and drafting the manuscript. NB: drafting the manuscript, interpretation of data, and finalizing it. RT, MB, RB-B, and MM-S: interpretation of data and drafting the manuscript. SA: contribution to the conception and design. SM: contribution to the conception and design of the work. AW: contribution to the revisions, interpretation of data, and drafting and editing final manuscript. All authors contributed to the article and approved the submitted version.

## Abbreviations

BMI: Body Mass Index
CP: Cinnamon polyphenols
DHEA: Dehydroepiandrosterone
ELISA: enzyme-linked immunosorbent assay
FAI: free androgen index
FBS: Fasting blood sugar
FSH: follicle-stimulating hormone
GLUT4: glucose transporter 4
HbA1c: Glycated hemoglobin
HDL-C: high-density lipoprotein
HMG-CoA: 3-hydroxy-3-methyl-glutaryl-CoA
HOMA-IR: Homeostatic Model Assessment for Insulin Resistance
IPAQ: international physical activity questionnaire
IUI: intrauterine insemination
LDL-C: low-density lipoprotein cholesterol
LH: Luteinizing hormone
PCOS: Polycystic ovary syndrome
PI3K: Phosphoinositide 3-kinases
SHBG: Sex hormone-binding globulin
TC: Total cholesterol
TG: triglyceride
WC: Waist Circumference.
