# Supplementary material for: A comparison of the effects of cinnamon, ginger, and metformin consumption on metabolic health, anthropometric indices, and sexual hormone levels in women with poly cystic ovary syndrome: A randomized double-blinded placebo-controlled clinical trial
Source: Front Nutr. 2022 Nov 29;9:1071515. doi: 10.3389/fnut.2022.1071515 (PMC9745082; doi:10.3389/fnut.2022.1071515)
Supplement: Supplementary file 1 [file Presentation_1.pptx]

## Slide 1
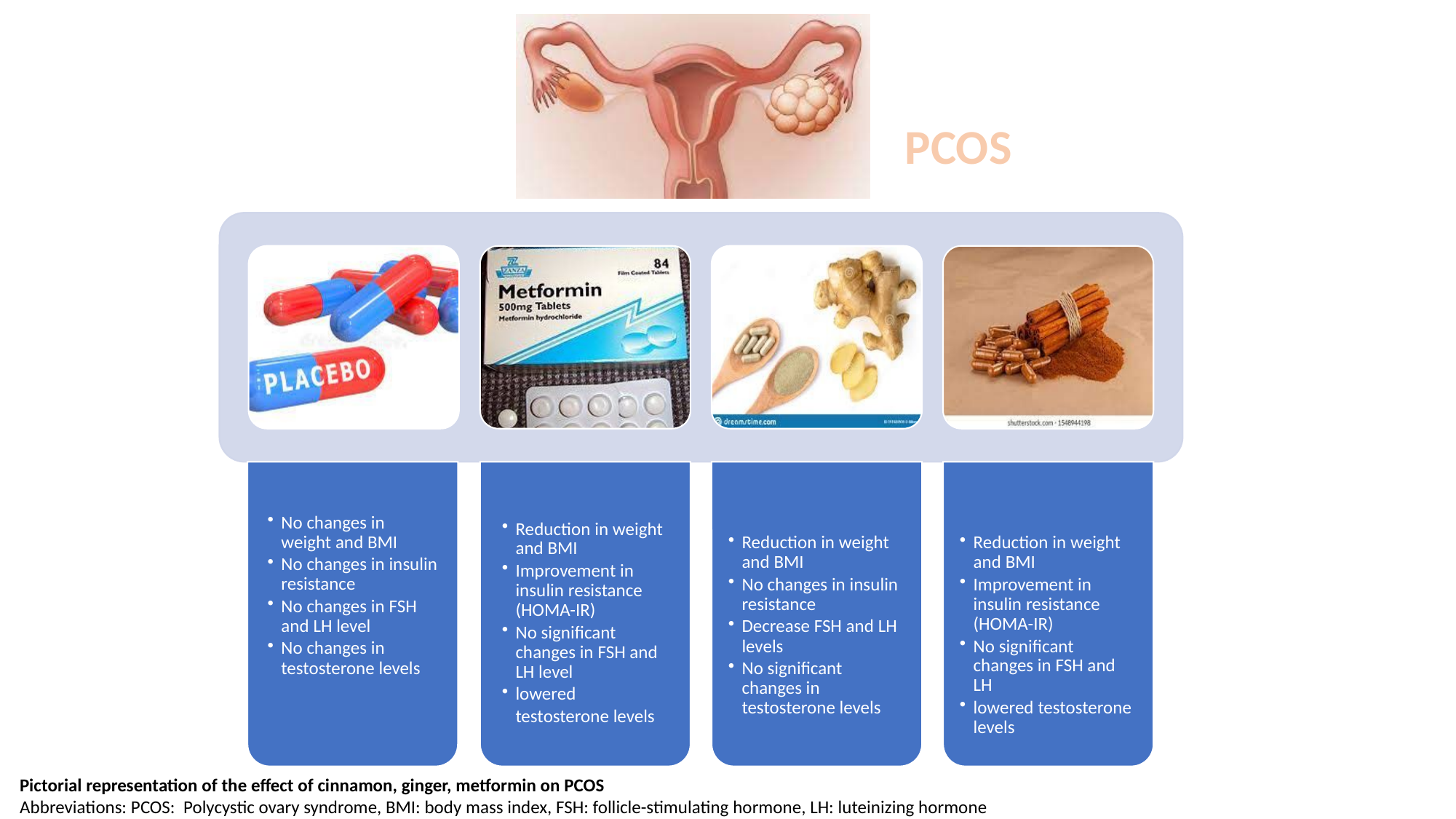

PCOS
Pictorial representation of the effect of cinnamon, ginger, metformin on PCOS
Abbreviations: PCOS: Polycystic ovary syndrome, BMI: body mass index, FSH: follicle-stimulating hormone, LH: luteinizing hormone
